# The gender- and age- dependent relationships between serum lipids and cognitive impairment: a cross-sectional study in a rural area of Xi’an, China

**DOI:** 10.1186/s12944-018-0956-5

**Published:** 2019-01-05

**Authors:** Beiyu Zhao, Suhang Shang, Pei Li, Chen Chen, Liangjun Dang, Yu Jiang, Jin Wang, Kang Huo, Meiying Deng, Jingyi Wang, Qiumin Qu

**Affiliations:** 1 Department of Neurology, First Affiliated Hospital of Xi’an Jiaotong University, 277 West Yanta Rd, Xi’an, 710061 China; 2 Huxian Hospital of Traditional Chinese Medicine, Xi’an, China

**Keywords:** Cognitive impairment, Risk factor, Serum lipid, Epidemiology

## Abstract

**Background:**

Serum lipids [total cholesterol (TC), low density lipoprotein cholesterol (LDL-C), high density lipoprotein cholesterol (HDL-C) and triglyceride (TG)] are risk factors for stroke, but the relationships between serum lipids and cognitive impairment have not been verified completely. In this study, we studied the relationships between serum lipids and cognitive impairment and explored whether gender and age had effects on the relationships.

**Methods:**

In this cross-sectional study, we collected serum lipids and cognitive function information from 1762 participants (aged 40–85). Univariate analysis, multivariate analysis, and both gender- and age-based stratified multivariate analysis were used.

**Results:**

In the entire sample set, there was no significant correlation between serum lipid parameters (TC, LDL-C, HDL-C and TG) and cognitive impairment. In both gender- and age-based stratified multivariate analysis, high serum TC was positively associated with cognitive impairment in the elderly (> 55) male participants (OR = 4.404, 95% CI = 1.264–15.344, *p* = 0.02), and high serum LDL-C was positively correlated with cognitive impairment in the elderly female subjects (OR = 2.496, 95% CI = 1.057–5.896, *p* = 0.037), while high serum TG was negatively associated with cognitive impairment in the middle-aged (≤ 55) male participants (OR = 0.157, 95% CI = 0.051–0.484, *p* = 0.001).

**Conclusions:**

The relationships between serum lipids and cognitive impairment are gender- and age- dependent, with high serum TC and LDL-C may be risk factors of cognitive impairment in the elderly male and female subjects respectively, while high serum TG may be protector of cognitive impairment in the middle-aged male participants.

## Introduction

Cognitive Impairment is emerging as one of the biggest health problems of the twenty-first century. There are nearly 46.8 million individuals living with cognitive impairment worldwide [1]. Cognitive impairment is a general term representing a group of disorders in which memory and thought courses become impaired, and it is frequently caused by Alzheimer’s disease (AD). Early intervention of subjects to prevent the onset of AD is particularly important because there is no efficient drug treatment available for AD.

With the development of economic level and the improvement of people’s living standards, the prevalence of dyslipidemia in China increased rapidly in the past ten years [2, 3]. Dyslipidemia, as a modifiable risk factor, is one of the vascular risk factors, and vascular disease is likely to have important effects on AD [4]. Some studies reported that this risk factor may also play a key role during the occurrence and development of AD [5, 6], but with conflicting results. One previous study showed a support for the hypothesis that higher levels of total cholesterol (TC) may induce the development of cognitive impairment [7]. Another research showed that lower level of TC may increase the risk of AD [8]. Conflicting results have also been found in studies relating low density lipoprotein cholesterol (LDL-C) [9, 10], high density lipoprotein cholesterol (HDL-C) [11, 12] and triglyceride (TG) [13, 14] levels with AD. Through the comparative analysis of relevant literatures, we speculate that the reason of contradictory results may be related to the different characteristics of study population and the incomprehensive possible influence factors which should be considered.

Previous studies showed that gender and age seem to be important factors in the association between serum lipids and cognitive function [15, 16], however, only a few studies have analyzed the relationship between serum lipids and cognitive impairment depending on both gender and age. So in order to explore the influence of gender and age on the relationship between serum lipids and cognitive impairment, more studies are necessary that include grouped subjects based on both gender and age. Because of different eating habits, low literacy, poor health consciousness and be indifferent to hyperlipidemia of Chinese people in rural areas, and while the previous studies were almost conducted in other countries, no similar research was carried on in rural areas of China, it is important to conduct such study.

Therefore, we studied the relationships between serum lipids and cognitive impairment from the middle-aged to the elderly (40–85) both in men and women. We explored that if dyslipidemia was correlated with cognitive impairment and if the correlation was regulated by both gender and age.

## Methods

### Study participants

In order to clarify the effect of serum lipid parameters on the cognitive impairment in Chinese of rural areas, participants were recruited from a village of Xi’an, from October 8, 2014, to March 30, 2015. This sample cohort included 1762 participants with age ≥ 40 years. Inclusion criteria are as follows: 1) permanent residents of the countryside, namely, the participants had the village’s household registration and had lived there for more than 3 years; 2) aged 40 or older; 3) agreed to join this research and be able to cooperate to complete the questionnaire survey, for illiterate participants, their relatives agreed on the informed consent.

Exclusion criteria are as follows: 1) individuals with a clear history of acute cerebrovascular disease, including stroke; 2) Individuals with diseases which could have an effect on cognitive function, excepting neurodegenerative disease or cerebrovascular disease, such as epilepsy (all types), brain trauma, nervous system tumor, past craniocerebral operation, congenital mental retardation, organic psychosis, affective psychosis, untreated hypothyroidism. 3) Individuals with serious liver or renal insufficiency. 4) Individuals who had malignant tumor or severe chronic disease. 5) Individuals who suffered from acute postoperative infection, inflammation, or major surgery. 6) Individuals who are taking lipid-lowering drugs, diuretics, sex hormones, oral contraceptives, glucocorticoids or immunosuppressant.

**Fig. 1:**
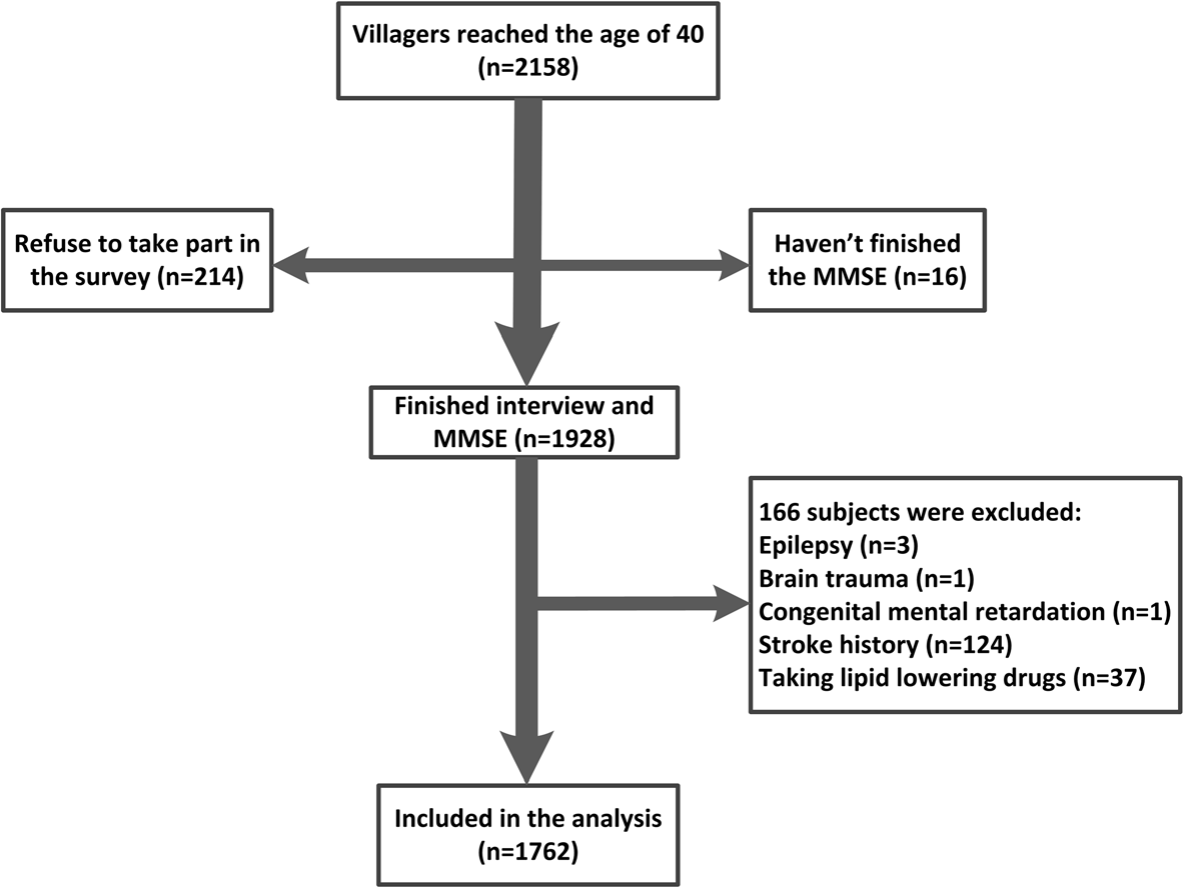
Flow chart. Abbreviations: MMSE, Mini-Mental State Examination

The selection of subjects and the study protocol are shown in Fig. 1. Every participant signed written informed consent, and the study was approved by the Medical Ethics Committee of the First Affiliated Hospital of Xi’an Jiaotong University.

### Cognitive evaluation

The Mini-Mental State Examination (MMSE) was used to evaluate the global cognition. Before the beginning of the study, every examiner accepted the unified training. According to the psychiatric rating scale defined by Ming-yuan Zhang et al. [17], we chose MMSE score lower than the boundary values as cognitive impairment criteria. This MMSE includes five parts: orientation (10 points), retention (3 points), attention and calculation (5 points), recall (3 points) and language (9 points), altogether 30 points. The boundary value was ≤17 for the uneducated, ≤ 20 for the primary school educated, and ≤ 24 for those accepted the junior high school or higher level of education.

### Serum lipids test

Five ml of elbow venous blood was taken from every participant in the morning on an empty stomach (8:00 am). Then the blood was centrifuged at 3000 G for 15 min and was rapidly stored at − 80 °C until later evaluation. The concentration of serum TC, LDL-C, HDL-C and TG levels were tested by enzymatic method using an automated biochemical analyzer (C501, Roche, Sweden). Quality indicators accord with the quality requirements proposed by the American National Cholesterol Educational Program. With reference to the diagnostic criteria of Chinese adult dyslipidemia prevention guide (2007 edition), dyslipidemia was defined as in according with at least one of the following components: high TC (≥ 5.18 mmol/L or receiving drug treatment for high TC); high LDL-C (≥ 3.37 mmol/L or receiving drug treatment for high LDL-C); low HDL-C (< 1.04 mmol/L or receiving drug treatment for low HDL-C) or high TG (≥ 1.70 mmol/L or receiving drug treatment for high TG) [18].

### Covariate

Covariates consisted of basic demographic information (gender, age, education years and marital status), life style (alcohol, tobacco, and physical exercise habits), comorbidities [hypertension (HP), diabetes mellitus (DM), coronary heart disease (CHD) and atrial fibrillation], family history (HP, DM, CHD, dyslipidemia), physical examination parameters [pulse rate, mean arterial pressure (MAP), cardiac murmur, body mass index (BMI) and arrhythmia] and biochemical indexes [fasting blood glucose level (FBG)]. It was defined as HP when systolic blood pressure (SBP) ≥ 140 mmHg and/or diastolic blood pressure (DBP) ≥ 90 mmHg [19]. MAP was calculated as 1/3 SBP plus 2/3 DBP. The diagnosis of DM was based on the WHO definition [20].

### Statistical analysis

All analyses were conducted using SPSS 13.0 statistical software. With double side inspection, *P* < 0.05 was considered significant. Graphs were plotted in GraphPad Prism version 5.0 for Windows. Data are given as mean ± standard deviation (SD) for continuous variables of normal distribution, as the median (25% percentile, 75% percentile) for continuous variables of skewed distribution or numerical value (percentage) for categorical variables. Differences between study groups were assessed using student t-tests for continuous variables of normal distribution, rank tests for continuous variables of skewed distribution, and χ^2^ tests for categorical variables.

As primary analysis, we showed the demographic and clinical data distribution. We conducted the statistical description about the distribution of general data characteristics, serum lipids, and possible factors affecting cognitive function.

In order to clarify the effects of gender and age on relationships between serum lipids and cognitive impairment, we conducted the following statistical analysis. First, we focused on the univariate analysis to explore the relationship between five serum lipid parameters and cognitive impairment respectively. In the process of analysis, the total population was divided into several subgroups respectively- women subgroup and men subgroup according to gender, the middle-aged (40–55) subgroup and the elderly (> 55) subgroup according to age [21], the middle-aged women subgroup, the elderly women subgroup, the middle-aged men subgroup and the elderly men subgroup according to both gender and age. We also analyzed the possible relationships between the confounders and cognitive dysfunction.

Second, on the basis of single factor analysis, we utilized multiple logistic regression analysis in all study subjects to adjust confounding factors and to further explain the effects of serum lipids on cognitive dysfunction. The inclusive criteria of covariates in multivariate analysis models are: factors that may probably influence cognitive function in previous studies, factors that may probably influence cognitive function which were focused on by this study, statistically significant factors in univariate analysis by this study. We also did interaction analysis of gender, age and serum lipids on cognitive impairment in the total population.

Furthermore, we did stratified analysis. The total population was divided into several subgroups based on gender, age, both gender and age respectively as mentioned above. In this part, we established three multivariate models to investigate if the relationship between serum lipid parameters and cognitive impairment is regulated by gender and age.

## Results

### Demographic and clinical data distribution

1762 people were actually included in the analysis of this study. 887 participants were diagnosed as having dyslipidemia. TC (5.05 ± 0.99 mmol/L), LDL-C (3.32 ± 0.89 mmol/L) and HDL-C (1.40 ± 0.31 mmol/L) were approximately normally distributed. TG [1.44 (1.02, 2.00) mmol/L] was skewed distributed. 226 subjects met the criteria of cognitive impairment and MMSE scores (25.66 ± 4.19) were normally distributed. Table 1 shows the demographic and general clinical information of the entire study sample set, as well as of subjects with and without dyslipidemia.

**Table 1:** Demographic and clinical characteristics of the participants (*n* = 1762)

Variables	Total (*n* = 1762)	Normal serum lipids(*n* = 875)	Dyslipidemia (*n* = 887)	*P*
Male [*n*(%)]	714(40.5)	364(41.6)	350(39.5)	0.360
Age [Mean(SD),year]	55.48(9.95)	54.60(10.03)	56.34(9.80)	< 0.001
Formal Education [*n*(%)]				0.009
Uneducated	224(12.7)	90(10.3)	134(15.1)	
Primary school	508(28.8)	263(30.1)	245(27.6)	
High school or above	1030(58.5)	522(59.7)	508(57.3)	
Education years [Median(P25, P75), y]	7(4.0,8.0)	7(4.3,8.0)	7(4.0,8.0)	0.042
Marital status [*n*(%)]				0.054
Married	1606(91.1)	809(92.5)	797(89.9)	
Others	156(8.9)	66(7.5)	90(10.1)	
Tobacco use [*n*(%)]	510(28.9)	267(30.5)	243(27.4)	0.149
Alcohol consumption [*n*(%)]	251(14.2)	124(14.2)	127(14.3)	0.930
Lack of physical activity [*n*(%)]	261(14.8)	121(13.8)	140(15.8)	0.248
Comorbidities [*n*(%)]				
HP	831(47.2)	351(40.1)	480(54.1)	0.001
DM	210(11.9)	72(8.2)	138(15.6)	0.001
CHD	38(2.2)	14(1.6)	24(2.7)	0.110
Atrial fibrillation	10(0.6)	2(0.2)	8(0.9)	0.060
Family history [*n*(%)]				
HP	542(30.8)	254(29.0)	288(32.5)	0.118
DM	142(8.1)	69(7.9)	73(8.2)	0.791
CHD	107(6.1)	57(6.5)	50(5.6)	0.441
Dyslipidemia	32(1.8)	15(1.7)	17(1.9)	0.751
Physical examination [*n*(%)]				
Cardiac murmur	28(1.6)	14(1.6)	14(1.6)	0.971
Arrhythmia	41(2.3)	27(3.1)	14(1.6)	0.136
MAP [Mean(SD), mmHg]	98.46(12.18)	95.99(11.55)	99.78(12.30)	< 0.001
Pulse Rate [Mean(SD)]	75.32(8.85)	75.29(8.64)	75.35(9.06)	0.818
BMI[Mean(SD), kg/m^2^]	25.28(3.15)	24.67(2.98)	25.88(3.31)	< 0.001
Biochemical examination				
FBG [Median(P25,P75), mmol/L]	5.40(5.06,5.82)	5.33(5.02,5.68)	5.49(5.12,6.00)	< 0.001
TC [Mean(SD), mmol/L]	5.05(0.99)	4.51(0.63)	5.58(0.99)	< 0.001
LDL [Mean(SD), mmol/L]	3.32(0.89)	2.86(0.65)	3.78(0.86)	< 0.001
HDL [Mean(SD), mmol/L]	1.40(0.31)	1.44(0.29)	1.37(0.33)	0.010
TG [Median(P25,P75), mmol/L]	1.44(1.02,2.00)	1.11(0.89,1.39)	1.99(1.49,2.53)	< 0.001
Cognition				
MMSE [Mean(SD)]	25.66(4.19)	25.87(4.15)	25.46(4.22)	0.038
Cognitive impairment [*n*(%)]	226(12.8)	101(11.5)	125(14.1)	0.110

*Abbreviations*, *SD* standard deviation; P25, 25% percentile; P75, 75% percentile, *HP* hypertension, *DM* diabetes mellitus, *CHD* coronary heart disease, *MAP* mean arterial pressure, *BMI* body mass index, *FBG* fasting blood glucose, *TC* total cholesterol, *LDL* low-density lipoprotein, *HDL* high-density lipoprotein, *TG* triglycerides, *MMSE* Mini-Mental State Examination

### Prevalence of cognitive impairment based on serum lipids in total population and subgroups

**Fig. 2:**
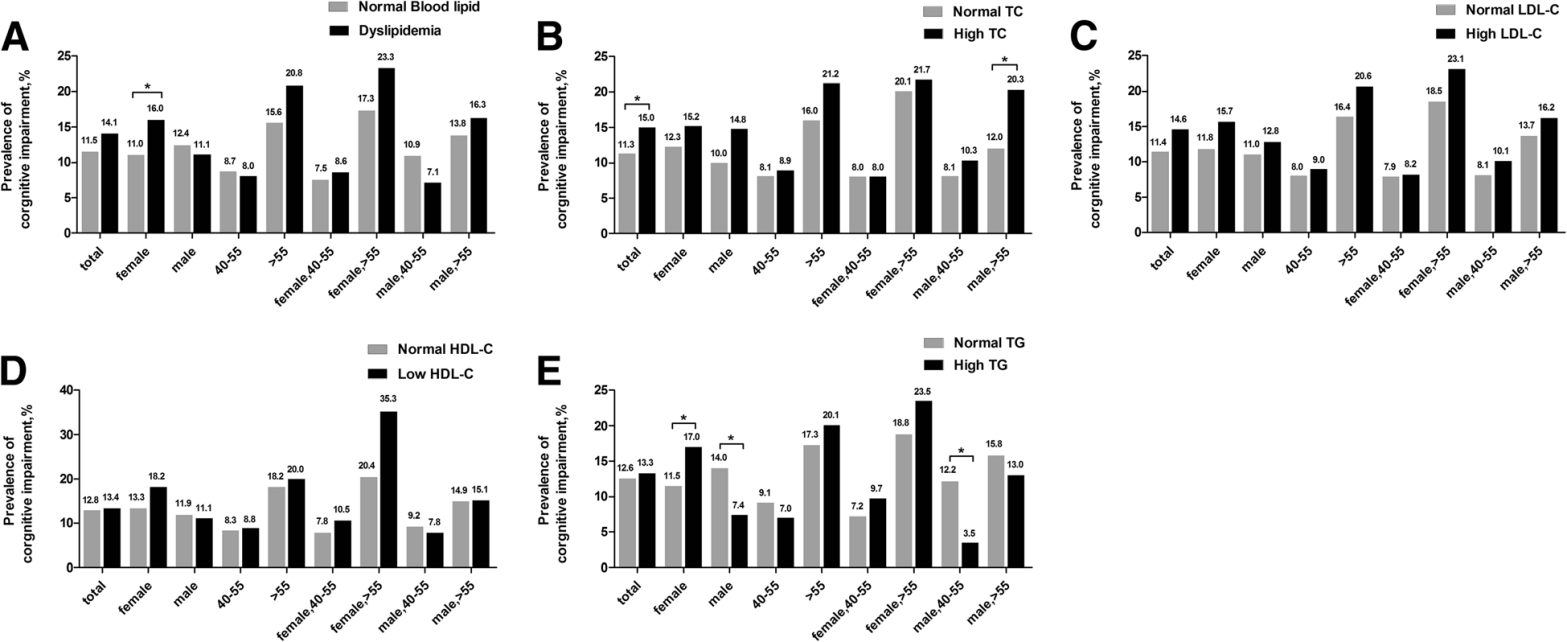
Prevalence of cognitive impairment based on dyslipidemia (**a**), TC (**b**), LDL-C (**c**), HDL-C (**d**) and TG (**e**) in total population and in the gender-based, age-based or gender- and age-based subgroups. * *P* < 0.05. Abbreviations: TC, total cholesterol; LDL, low-density lipoprotein; HDL, high-density lipoprotein; TG, triglycerides

Figure 2 shows the prevalence of cognitive impairment. In the total population, the prevalence of cognitive impairment was no significantly different between dyslipidemia group and normal lipid group, but in the female subgroup, the prevalence of cognitive impairment in the dyslipidemia group was higher than that in the normal lipid group (Fig. 2a).

In total population, the prevalence of cognitive impairment in the high TC group was higher than that in the normal TC group significantly (Fig. 2b). Gender- and age-stratified univariate analysis showed that in the elderly (> 55) male subgroup the prevalence of cognitive impairment was significantly higher in the high TC group than that in the normal TC group (Fig. 2b). In total population, the prevalence of cognitive impairment had no significant difference between the normal LDL-C group and the high LDL-C group (Fig. 2c), neither between the normal HDL-C group and the low HDL-C group (Fig. 2d). After stratified by both gender and age, the prevalence of cognitive impairment was still no significantly different between the normal LDL-C group and the high LDL-C group, nor between the normal HDL-C group and the low HDL-C group. In total population, the prevalence of cognitive impairment was no significantly different between the high TG group and the normal TG group, but in the female subgroup, the prevalence of cognitive impairment in the high TG group was higher than that in the normal TG group, while in the male subgroup, the prevalence of cognitive impairment in the high TG group was lower than that the in normal TG group (Fig. 2e). Table 2 shows differences in covariates, such as gender, age, education years and so on, between the cognitive impairment groups and the normal cognition groups.

**Table 2:** Differences in covariates between the cognitive impairment group and the normal cognition group

Variables	CI (*n* = 226)	NC (*n* = 1536)	*P*
Gender [*n*(%)]			0.271
Male	84(11.8)	630(88.2)	
Female	142(13.5)	906(86.5)	
Age [*n*(%)]			< 0.001
40–55	82(8.4)	897(91.6)	
> 55	144(18.4)	639(81.6)	
Education years [Median(P25,P75), y]	8(5.0,9.0)	5(0.0,8.0)	< 0.001
Marital status [*n*(%)]			< 0.001
Married	190(11.8)	1416(88.2)	
Others	36(23.1)	120(76.9)	
Tobacco use [*n*(%)]			0.704
Y	63(12.4)	447(87.6)	
N	163(13.0)	1089(87.0)	
Alcohol consumption [*n*(%)]			0.207
Y	26(10.4)	225(89.6)	
N	200(13.2)	1311(86.8)	
Lack of physical activity [*n*(%)]			0.021
Y	45(17.2)	216(82.8)	
N	181(12.1)	1320(87.9)	
HP [*n*(%)]			0.013
Y	124(14.9)	707(85.1)	
N	102(11.0)	829(89.0)	
DM [*n*(%)]			0.001
Y	42(20.0)	168(80.0)	
N	184(11.9)	1368(88.1)	
CHD [*n*(%)]			0.006
Y	11(28.9)	27(71.1)	
N	215(12.5)	1509(87.5)	
Atrial fibrillation [*n*(%)]			0.837
Y	2(20.0)	8(80.0)	
N	224(12.8)	1528(87.2)	
Family history of HP [*n*(%)]			< 0.001
Y	43(7.9)	499(92.1)	
N	182(15.0)	1034(85.0)	
U	1(25.0)	3(75.0)	
Family history of DM [*n*(%)]			0.701
Y	15(10.6)	127(89.4)	
N	210(13.0)	1402(87.0)	
vU	1(12.5)	7(87.5)	
Family history of CHD [*n*(%)]			0.034
Y	5(4.7)	102(95.3)	
N	220(13.3)	1428(86.7)	
U	1(14.3)	6(85.7)	
Family history of Dyslipidemia [*n*(%)]			0.529
Y	2(6.3)	30(93.7)	
N	223(12.9)	1500(87.1)	
U	1(14.3)	6(85.7)	
Cardiac murmur [*n*(%)]			0.034
Y	8(28.6)	20(71.4)	
N	217(12.6)	1502(87.4)	
U	1(6.7)	14(93.3)	
Arrhythmia [*n*(%)]			0.127
Y	9(22.0)	32(78.0)	
N	217(12.7)	1497(87.3)	
U	0(0.0)	7(100.0)	
MAP [Mean(SD), mmHg]	100.90(12.71)	98.10(12.06)	0.002
Pulse Rate [Mean(SD)]	76.13(10.67)	75.20(8.55)	0.321
BMI[Mean(SD), kg/m^2^]	24.83(3.359)	25.35(3.181)	0.024
FBG [Median(P25,P75), mmol/L]	5.39(5.07,5.80)	5.42(5.04,6.02)	0.394

*Abbreviations*, CI cognitive impairment, *NC* normal cognition, P25, 25% percentile, P75, 75% percentile, *Y* yes, *N* no, *U* unknown, *HP* hypertension, *DM* diabetes mellitus, *CHD* coronary heart disease, *SD* standard deviation, *MAP* mean arterial pressure, *BMI* body mass index, *FBG* fasting blood glucose

### Multivariate analysis of serum lipids on cognitive impairment in the total population

**Fig. 3:**
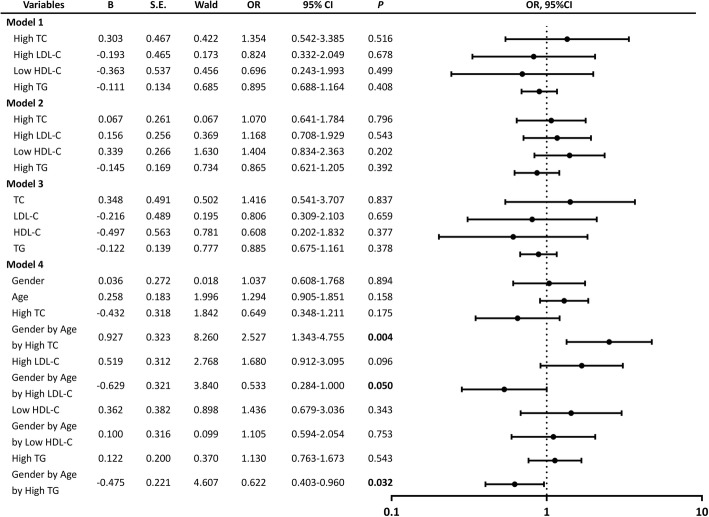
Forest plot of relationships between serum lipid parameters and cognitive impairment in the total population with multivariate analysis (model 1, 2, 3 and 4). High TC, high LDL-C, low HDL-C and high TG were treated as binary data (yes or no) in model 1 and 2. In model 3, TC, LDL-C, HDL-C and TG were transformed into continuous variables. In model 1, the analysis was corrected for gender, age, and years of education. Model 2 was adjusted for the covariates included in model 1 as well as for tobacco use, alcohol consumption, lack of physical activity, CHD, MAP, BMI, FBG. In model 3, the confounding variables considered were the same as those considered in model 2. Model 4 was adjusted for the covariates included in model 2 plus the interaction terms gender by age by serum lipids. Abbreviations: OR, odds ratio; CI, confidence interval; TC, total cholesterol; LDL, low-density lipoprotein; HDL, high-density lipoprotein; TG, triglycerides; CHD, coronary heart disease; MAP, mean arterial pressure; BMI, body mass index; FBG, fasting blood glucose

To explore relationships between serum lipids and cognitive impairment further, we conducted multiple logistic regression analysis in all study subjects (Fig. 3). Multivariate analysis showed that controlling for possible confounding factors, there was no significant correlation between high TC, high LDL-C, low HDL-C or high TG with cognitive impairment (models 1 and 2). As well, no significant correlation was found between TC, LDL-C, HDL-C, TG and cognitive impairment (model 3). The hypothesis test of regression coefficients for all covariates in model 2 has been did and the results showed that the effect on high TC, high LDL-C, age, education years, lack of physical activity, CHD, BMI and MAP on cognitive impairment was significant, while the effect of other covariates had no significance on cognition (data not shown). Next we established model 4 to examine the interaction of serum lipid parameters (high TC, high LDL-C, low HDL-C and high TG), gender and age. The interaction term gender by age by high TC was positively correlated with cognitive impairment (OR = 2.236, *P* = 0.009), the interaction term gender by age by high LDL-C was negatively correlated with cognitive impairment (OR = 0.533, *P* = 0.041), meanwhile, the effect of the interaction of gender, age and high TG on cognitive impairment was similar to that of gender, age and high LDL-C on cognitive impairment. It indicated that gender and age had effects on the relationship between serum lipid parameters and cognitive impairment.

### Multivariate analysis of relationships between serum lipids and cognitive impairment in subgroups stratified by gender and age

**Fig. 4:**
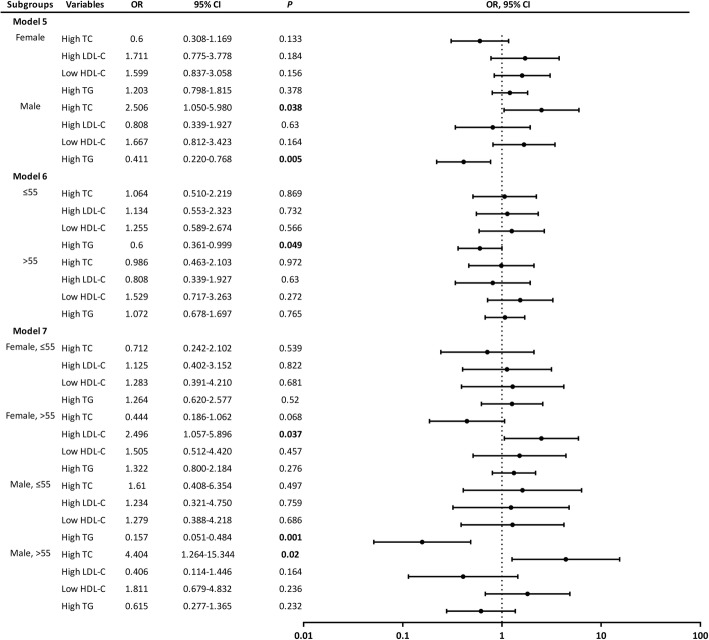
Forest plot of relationships between serum lipid parameters and cognitive impairment in the subgroups stratified by gender and age (models 5, 6 and 7). The population was divided into two gender-based subgroups (male and female) in model 5, two age-based subgroups (≤ 55 and > 55) in model 6, four both gender and age-based subgroups (female, ≤ 55; female, > 55; male, ≤ 55 and male, > 55) in model 7. Every serum lipid parameter (high TC, high LDL-C, low HDL-C and high TG) was included in every subgroup of these 3 models. The confounding variables considered in model 5, 6 and 7 were as follows: years of education, tobacco use, alcohol consumption, lack of physical activity, CHD, MAP, BMI, and FBG. Abbreviations: OR, odds ratio; CI, confidence interval; TC, total cholesterol; LDL, low-density lipoprotein; HDL, high-density lipoprotein; TG, triglycerides; CHD, coronary heart disease; MAP, mean arterial pressure; BMI, body mass index; FBG, fasting blood glucose

To illustrate the effect of both gender and age on relationships between serum lipids and cognitive impairment further, stratified analysis was used (Fig. 4, models 5, 6 and 7). Confounders introduced into models 5, 6 and 7 were the same as those considered in model 2. In model 5, the stratified logistic regression results showed that high TC related with cognitive impairment positively (OR = 2.506, *p* = 0.038) in the male subgroup, while high TG associated with cognitive impairment negatively (OR = 0.411, *p* = 0.005) in the same subgroup. However, there was no difference of the other serum lipids and cognitive impairment in model 5. In model 6, high TG associated with cognitive impairment negatively (OR = 0.600, *p* = 0.049) in the middle-aged subgroup. Age stratified multivariate analysis showed that there seemed to be no difference between the other serum lipid indexes and cognitive impairment. Both gender and age stratified multivariate analysis showed that high LDL-C associated with cognitive impairment positively (OR = 2.496, *p* = 0.037) in the elderly female subgroup, while high TG associated with cognitive impairment negatively (OR = 0.157, *p* = 0.001) in the middle-aged male subgroup and high TC associated with cognitive impairment positively (OR = 4.404, *p* = 0.02) in the elderly male subgroup. However, no significant correlation between the other serum lipid parameters and cognitive impairment was found in model 7.

## Discussion

In this cross-sectional study, we found there was no association between serum lipids and cognitive impairment in the total population. However, after stratified according to gender and age, with adjustment for confounding factors, the correlation ships between serum lipids and cognitive impairment were prominent. In the elderly male participants, there was a positive association between serum TC and cognitive impairment, while among the elderly female subjects, the correlation was positive between serum LDL-C and cognitive impairment. In the middle-aged male participants, the correlation ship was negative between TG and cognitive impairment. However, the data showed no association between HDL-C and cognitive impairment. The results above demonstrated that relationships between serum lipids and cognitive impairment were both gender- and age-dependent.

TC is the most important lipids, and transported by LDL-C and HDL-C in the blood mainly. A previous study showed that high serum TC level could lead to bearing an increased risk of AD in old participants [15], while another study found the association was only in the middle-aged people [22], and one study reported that the association was only found in male participants [23]. One study found that high LDL-C was negatively correlated with cognitive impairment [24], one showed the correlation was opposite [25]. The relationship between serum HDL-C levels and cognitive impairment remained unclear [26, 27]. One suggested an association between lower HDL-C levels and poor cognitive function [28], while another reported that HDL-C levels were not related to cognitive function [29]. Therefore, results of the previous researches showed that the relationship between cholesterol and cognitive impairment still remained unclear. So in order to explore the clear and deep relationship between dyslipidemia and cognitive impairment, stratification analysis according to gender and age was conducted in this study. Participants were recruited from a village of Xi’an, and after stratified according to gender and age, we found that TC and LDL-C levels were positively associated with cognitive impairment in the elderly male and elderly female participants respectively, while HDL-C level was not associated with cognitive function. These results indicated that TC and LDL-C levels related to cognitive impairment were gender- and age- dependent.

The mechanism of dyslipidemia, particularly high TC and high LDL-C level, increasing the risk of cognitive impairment is still unclear at present. However, high concentrations of TC and LDL-C have been proven to be independent risk factors of cerebrovascular diseases [30], which in turn may result in cognitive impairment through cerebral ischemia. Furthermore, leucoaraiosis, which belongs to the pathological change of cerebrovascular diseases, is a progressive process that is associated with cognitive impairment [31]. High TC and LDL-C levels may also be connected with small-vessel diseases by holding a vital role in maintaining the balance of cholesterol levels in the brain. Thus, high TC and LDL-C could promote cognitive impairment through a cerebrovascular pathway. Meanwhile, it is possible that high concentrations of TC and high LDL-C could affect cognitive function through non-cerebrovascular mechanisms. Animal study provided proofs for the function of cholesterol in the production of amyloid-β (Aβ), a hallmark of AD pathogenesis, by demonstrating that a high cholesterol diet sustaining for 7–10 months could increase TC level by 4-fold and increase Aβ accumulation in mice [32]. This conclusion has also been demonstrated by the rabbit experiment [33]. Experimental study found that cholesterol reduction could lower the production of Aβ [34]. Also, there was evidence that larger amount of Aβ deposition was associated with high TC and LDL-C levels measured during lifetime in autopsied brains from AD patients [35]. Furthermore, previous studies showed that cholesterol increased the activity of β-secretase, which is important for activity of the amyloid precursor protein secretase, and implied that high cholesterol levels could increase Aβ generation [36, 37]. Based on these findings, we can conjecture that high levels of TC and LDL-C contribute to cognitive impairment by cerebral hypoperfusion, white matter injuries, the disorder of transport in cerebrovascular pathway, and by increasing the deposition of Aβ and the activity of β-secretase in non-cerebrovascular pathway.

Why TC and LDL-C related to cognitive impairment only in elderly people? One possible reason is that it takes a long time for high serum TC and LDL-C levels to induce atherosclerosis, small vessel lesions of the brain capillaries, and increase the deposition of Aβ and the activity of β-secretase in both cerebrovascular and non-cerebrovascular pathways, which contribute to the development of AD [38]. The elderly themselves are vulnerable to hyperlipidemia may be another possible reason. To demonstrate if high TC levels in the middle-aged associate with an increased risk of cognitive impairment in old age, cohort studies are needed. Moreover, our study showed that the positive correlation between TC levels and cognitive impairment was only in males, while the association was only in females when referred to LDL-C levels. This gender difference may be related to different hormonal status. Estrogen which could modify the cholesterol metabolic process was lower in postmenopausal women [39]. Animal study has suggested sex differences linked to a difference in LDL receptor [40]. A previous study found that higher TC related to increase cortical thinning, which related to AD, was only in male population [41].

TG is another form of serum lipids. Several previous studies have reported that there was no association between TG levels and AD risk [42, 43], similarly, our study demonstrated no significant association between TG levels and cognitive impairment in entire sample set, however, after stratified according to gender and age, high TG was negatively correlated with cognitive impairment in the middle-aged male participants. Low TG concentrations were correlated with the brain inflammation, frailty and low nutrition levels [44]. Low TG levels may in turn reflect a low nutrition level diet implying pathological changes or be a marker for early cognitive impairment. However, we don’t know any reasonable physiologic progression for such a causal relationship clearly. Moreover, the reasons for this inconsistency in different genders could partly be attributed to the truth that cognitive impairment is quite different condition with gender differences [45].

Several limitations of this research should be noted. Firstly, one of limitations of the present study is that it is a cross-sectional study, and so no causal relationships between serum lipids and cognitive impairment could be reached. Secondly, because of different eating habits, low literacy, poor health consciousness and be indifferent to hyperlipidemia of Chinese people in rural areas, and while the previous studies were almost conducted in other countries, no similar research was carried on in rural areas of China, so we conducted this study. Actually, the participants with dyslipidemia in this study were significantly older, had a lower education level, and had a higher rate of comorbidities compared to normolipidemic participants, and these confounding factors themselves could significantly increase the risk of CI, so we adjusted confounding factors and did stratified analysis to illustrate the effect of both gender and age on relationships between serum lipids and cognitive impairment further. Thirdly, we evaluated cognition only using the MMSE. Although the MMSE is a convenient measurement of global cognition and has been used in previous studies widely, but it should be note that MMSE has false positive and false negative. The cognitive impairment diagnosed by MMSE needs to be confirmed by other neuropsychiatric test. Meanwhile, we did not determine apolipoprotein-E genotypes, which is the most powerful genetic dangerous factor of AD. As apolipoprotein-E also associated with serum lipids transport, it may have impact on relationships between serum lipid parameters and cognitive impairment.

In conclusion, this present study suggested that relationships between serum lipids and cognitive impairment are gender- and age- dependent, with high serum TC and high LDL-C may be risk factors for cognitive impairment in the elderly male and elderly female subjects respectively, while high serum TG may be protector of cognitive impairment in the middle-aged male participants. Additional large longitudinal studies with comprehensive measurements of cognitive function are required.

## References

[1] Prince M, Wimo A, Guerchet M, Ali GC, Wu YT, Prina M, et al. World Alzheimer Report 2015: The Global Impact of Dementia. London: Alzheimer’s Disease International; 2015.

[2] Cai L, Zhang L, Liu A, Li S, Wang P (2012). Prevalence, awareness, treatment, and control of dyslipidemia among adults in Beijing, China. J Atheroscler Thromb.

[3] Qi L, Ding X, Tang W, Li Q, Mao D, Wang Y (2015). Prevalence and risk factors associated with dyslipidemia in Chongqing, China. Int J Environ Res Public Health.

[4] Luchsinger JA, Reitz C, Honig LS, Tang MX, Shea S, Mayeux R (2005). Aggregation of vascular risk factors and risk of incident Alzheimer disease. Neurology.

[5] Reis JP, Loria CM, Launer LJ, Sidney S, Liu K, Jacobs DR, Zhu N, Lloyd-Jones DM, He K, Yaffe K (2013). Cardiovascular health through young adulthood and cognitive functioning in midlife. Ann Neurol.

[6] Zeki Al Hazzouri A, Haan MN, Neuhaus JM, Pletcher M, Peralta CA, Lopez L, Perez Stable EJ (2013). Cardiovascular risk score, cognitive decline, and dementia in older Mexican Americans: the role of sex and education. J Am Heart Assoc.

[7] Ledreux A, Wang X, Schultzberg M, Granholm AC, Freeman LR (2016). Detrimental effects of a high fat/high cholesterol diet on memory and hippocampal markers in aged rats. Behav Brain Res.

[8] Tukiainen T, Jylanki P, Makinen VP, Grohn O, Hallikainen M, Soininen H, Kivipelto M, Kaski K, Groop PH, Savolainen MJ (2012). Mild cognitive impairment associates with concurrent decreases in serum cholesterol and cholesterol-related lipoprotein subclasses. J Nutr Health Aging.

[9] Gonzalez-Escamilla G, Atienza M, Garcia-Solis D, Cantero JL (2016). Cerebral and blood correlates of reduced functional connectivity in mild cognitive impairment. Brain Struct Funct.

[10] Ma C, Li J, Bao Z, Ruan Q, Yu Z (2015). Serum levels of ApoA1 and ApoA2 are associated with cognitive status in older men. Biomed Res Int.

[11] Solfrizzi V, Scafato E, Capurso C, D'Introno A, Colacicco AM, Frisardi V, Vendemiale G, Baldereschi M, Crepaldi G, Di Carlo A (2010). Metabolic syndrome and the risk of vascular dementia: the Italian longitudinal study on ageing. J Neurol Neurosurg Psychiatry.

[12] Leritz EC, McGlinchey RE, Salat DH, Milberg WP (2016). Elevated levels of serum cholesterol are associated with better performance on tasks of episodic memory. Metab Brain Dis.

[13] Hall K, Murrell J, Ogunniyi A, Deeg M, Baiyewu O, Gao S, Gureje O, Dickens J, Evans R, Smith-Gamble V (2006). Cholesterol, APOE genotype, and Alzheimer disease: an epidemiologic study of Nigerian Yoruba. Neurology.

[14] Koyama A, Stone K, Yaffe K (2013). Serum oxidized low-density lipoprotein level and risk of cognitive impairment in older women. Neurobiol Aging.

[15] Toro P, Degen C, Pierer M, Gustafson D, Schroder J, Schonknecht P (2014). Cholesterol in mild cognitive impairment and Alzheimer's disease in a birth cohort over 14 years. Eur Arch Psychiatry Clin Neurosci.

[16] Reynolds CA, Gatz M, Prince JA, Berg S, Pedersen NL (2010). Serum lipid levels and cognitive change in late life. J Am Geriatr Soc.

[17] Katzman R, Zhang MY, Ouang Ya Q, Wang ZY, Liu WT, Yu E, Wong SC, Salmon DP, Grant I (1988). A Chinese version of the mini-mental state examination; impact of illiteracy in a Shanghai dementia survey. J Clin Epidemiol.

[18] Joint Committee for Developing Chinese guidelines on Prevention and Treatment of Dyslipidemia in Adults. Chinese guidelines on prevention and treatment of dyslipidemia in adults. Zhonghua Xin Xue Guan Bing Za Zhi. 2007;35:390–419. 17711682

[19] James PA, Oparil S, Carter BL, Cushman WC, Dennison-Himmelfarb C, Handler J, Lackland DT, LeFevre ML, MacKenzie TD, Ogedegbe O (2014). 2014 evidence-based guideline for the management of high blood pressure in adults: report from the panel members appointed to the eighth joint National Committee (JNC 8). JAMA.

[20] Alberti KG, Zimmet PZ (1998). Definition, diagnosis and classification of diabetes mellitus and its complications. Part 1: diagnosis and classification of diabetes mellitus provisional report of a WHO consultation. Diabet Med.

[21] Li S, Li L, Ai X, Yang L, Bai Y, Wang Z, Han H, Lu Q, Luo F, Zhang Z (2014). A randomized, controlled, blinded study of the safety, immunogenicity and batch consistency of aleph inactivated split influenza vaccine made in China in Chinese people. Hum Vaccin Immunother.

[22] Kivipelto M, Ngandu T, Fratiglioni L, Viitanen M, Kareholt I, Winblad B, Helkala EL, Tuomilehto J, Soininen H, Nissinen A (2005). Obesity and vascular risk factors at midlife and the risk of dementia and Alzheimer disease. Arch Neurol.

[23] Ancelin ML, Ripoche E, Dupuy AM, Samieri C, Rouaud O, Berr C, Carriere I, Ritchie K (2014). Gender-specific associations between lipids and cognitive decline in the elderly. Eur Neuropsychopharmacol.

[24] Scacchi R, De Bernardini L, Mantuano E, Vilardo T, Donini LM, Ruggeri M, Gemma AT, Pascone R, Corbo RM (1998). DNA polymorphisms of apolipoprotein B and angiotensin I-converting enzyme genes and relationships with lipid levels in Italian patients with vascular dementia or Alzheimer's disease. Dement Geriatr Cogn Disord.

[25] Li L, Cao D, Desmond R, Rahman A, Lah JJ, Levey AI, Zamrini E (2008). Cognitive performance and plasma levels of homocysteine, vitamin B12, folate and lipids in patients with Alzheimer disease. Dement Geriatr Cogn Disord.

[26] Muckle TJ, Roy JR (1985). High-density lipoprotein cholesterol in differential diagnosis of senile dementia. Lancet.

[27] Wieringa GE, Burlinson S, Rafferty JA, Gowland E, Burns A (1997). Apolipoprotein E genotypes and serum lipid levels in Alzheimer's disease and multi-infarct dementia. Int J Geriatr Psychiatry.

[28] Szoeke C, Lehert P, Henderson VW, Dennerstein L, Desmond P, Campbell S (2016). Predictive factors for verbal memory performance over decades of aging: data from the Women's healthy ageing project. Am J Geriatr Psychiatry.

[29] Goh VH, Hart WG (2014). The association of metabolic syndrome and aging with cognition in Asian men. Aging Male.

[30] Sharrett AR, Patsch W, Sorlie PD, Heiss G, Bond MG, Davis CE (1994). Associations of lipoprotein cholesterols, apolipoproteins A-I and B, and triglycerides with carotid atherosclerosis and coronary heart disease. The atherosclerosis risk in communities (ARIC) study. Arterioscler Thromb.

[31] Schmidt R, Ropele S, Enzinger C, Petrovic K, Smith S, Schmidt H, Matthews PM, Fazekas F (2005). White matter lesion progression, brain atrophy, and cognitive decline: the Austrian stroke prevention study. Ann Neurol.

[32] Shie FS, Jin LW, Cook DG, Leverenz JB, LeBoeuf RC (2002). Diet-induced hypercholesterolemia enhances brain a beta accumulation in transgenic mice. Neuroreport.

[33] Levin-Allerhand JA, Lominska CE, Smith JD (2002). Increased amyloid- levels in APPSWE transgenic mice treated chronically with a physiological high-fat high-cholesterol diet. J Nutr Health Aging.

[34] Refolo LM, Pappolla MA, LaFrancois J, Malester B, Schmidt SD, Thomas-Bryant T, Tint GS, Wang R, Mercken M, Petanceska SS, Duff KE (2001). A cholesterol-lowering drug reduces beta-amyloid pathology in a transgenic mouse model of Alzheimer's disease. Neurobiol Dis.

[35] Kuo YM, Emmerling MR, Bisgaier CL, Essenburg AD, Lampert HC, Drumm D, Roher AE (1998). Elevated low-density lipoprotein in Alzheimer's disease correlates with brain abeta 1-42 levels. Biochem Biophys Res Commun.

[36] Cordy JM, Hussain I, Dingwall C, Hooper NM, Turner AJ (2003). Exclusively targeting beta-secretase to lipid rafts by GPI-anchor addition up-regulates beta-site processing of the amyloid precursor protein. Proc Natl Acad Sci U S A.

[37] Refolo LM, Malester B, LaFrancois J, Bryant-Thomas T, Wang R, Tint GS, Sambamurti K, Duff K, Pappolla MA (2000). Hypercholesterolemia accelerates the Alzheimer's amyloid pathology in a transgenic mouse model. Neurobiol Dis.

[38] Ehrlich D, Humpel C (2012). Chronic vascular risk factors (cholesterol, homocysteine, ethanol) impair spatial memory, decline cholinergic neurons and induce blood-brain barrier leakage in rats in vivo. J Neurol Sci.

[39] Dupuy AM, Carriere I, Scali J, Cristol JP, Ritchie K, Dartigues JF, Gambert P, Ancelin ML (2008). Lipid levels and cardiovascular risk in elderly women: a general population study of the effects of hormonal treatment and lipid-lowering agents. Climacteric.

[40] Segatto M, Trapani L, Marino M, Pallottini V (2011). Age- and sex-related differences in extra-hepatic low-density lipoprotein receptor. J Cell Physiol.

[41] Walhovd KB, Storsve AB, Westlye LT, Drevon CA, Fjell AM (2014). Blood markers of fatty acids and vitamin D, cardiovascular measures, body mass index, and physical activity relate to longitudinal cortical thinning in normal aging. Neurobiol Aging.

[42] Dupuy AM, Mas E, Ritchie K, Descomps B, Badiou S, Cristol JP, Touchon J (2001). The relationship between apolipoprotein E4 and lipid metabolism is impaired in Alzheimer's disease. Gerontology.

[43] Czyzewski K, Lalowski MM, Pfeffer A, Barcikowska M (2001). Lipid metabolism parameters in patients with Alzheimer's disease and their first degree relatives. Acta Neurobiol Exp (Wars).

[44] Hu P, Seeman TE, Harris TB, Reuben DB (2003). Does inflammation or undernutrition explain the low cholesterol-mortality association in high-functioning older persons? MacArthur studies of successful aging. J Am Geriatr Soc.

[45] Ritchie K, Ancelin ML, Beaino E, Portet F, Brickman AM, Dartigues JF, Tzourio C, Dupuy AM, Ritchie CW, Berr C, Artero S (2010). Retrospective identification and characterization of mild cognitive impairment from a prospective population cohort. Am J Geriatr Psychiatry.

